# Understanding Primary Blast Injury: High Frequency Pressure Acutely Disrupts Neuronal Network Dynamics in Cerebral Organoids

**DOI:** 10.1089/neu.2022.0044

**Published:** 2022-11-01

**Authors:** Marc Joshua Silvosa, Nohemi Romo Mercado, Nikolas Merlock, Suhas Vidhate, Ricardo Mejia-Alvarez, Tony T. Yuan, Adam M. Willis, Zane R. Lybrand

**Affiliations:** ^1^ Division of Biology, Texas Woman's University, Denton, Texas, USA.; ^2^ Department of Neuroscience, Developmental and Regenerative Biology, University of Texas at San Antonio, San Antonio, Texas, USA.; ^3^ Department of Mechanical Engineering, Michigan State University, East Lansing, Michigan, USA.; ^4^ Department of Radiology and Imaging Sciences, National Institutes of Health, Bethesda, Maryland, USA.; ^5^ 59th Medical Wing Science and Technology, Joint Base San Antonio-Lackland, Texas, USA.

**Keywords:** blast, high frequency pressure waves, organoid, pluripotent stem cell, traumatic brain injury

## Abstract

Blast exposure represents a common occupational risk capable of generating mild to severe traumatic brain injuries (TBI). During blast exposure, a pressure shockwave passes through the skull and exposes brain tissue to complex pressure waveforms. The primary neurophysiological response to blast-induced pressure waveforms remains poorly understood. Here, we use a computer-controlled table-top pressure chamber to expose human stem cell–derived cerebral organoids to varied frequency of pressure waves and characterize the neurophysiological response. Pressure waves that reach a maximum amplitude of 250 kPa were used to model a less severe TBI and 350 kPa for a more severe blast TBI event. With each amplitude, a frequency range of 500 Hz, 3000 Hz, and 5000 Hz was tested. Following the 250 kPa overpressure a multi-electrode array recorded organoid neural activity. We observed an acute suppression neuronal activity in single unit events, population events, and network oscillations that recovered within 24 h. Additionally, we observed a network desynchronization after exposure higher frequency waveforms. Conversely, organoids exposed to higher amplitude pressure (350k Pa) displayed drastic neurophysiological differences that failed to recover within 24 h. Further, lower amplitude “blast” (250 kPa) did not induce cellular damage whereas the higher amplitude “blast” (350 kPa) generated greater apoptosis throughout each organoid. Our data indicate that specific features of pressure waves found intracranially during blast TBI have varied effects on neurophysiological activity that can occur even without cellular damage.

## Introduction

Blast-induced traumatic brain injury (TBI) is ubiquitous across the modern combat and civilian environments. During Operational Enduring Freedom and Iraqi Freedom (OEF/OIF), approximately 320,000 personnel had some level of TBI, with 52% attributed to improvised explosive devices.^1^ Additionally, of all hospitalized U.S. soldiers from Iraq and Afghanistan, explosions accounted for over 65% of admissions for TBI.^2^ Many civilian occupations that include demolition, construction, and the operation of heavy machinery in industries like oil and gas and commercial airlines are exposed to variable levels of blast exposures. How this affects human health and brain function, especially with regard to low-level blast (LLB) remains largely unexplored.^3^ Blast exposures are categorized into primary (effects directly from pressure waves), secondary (effects from material propelled by shock waves), tertiary (whole–body accelerations/decelerations), and quaternary/miscellaneous (burns, electromagnetic, toxins, etc.) injuries. Although secondary to quaternary injuries have defined parameters, it remains a significant challenge in determining primary injury (pressure) effects at cellular and tissue levels. This will be crucial in defining risk thresholds for blast exposure, as well as understanding blast TBI physiology that supports personnel protective equipment (PPE) improvement and the development of pharmaceutical countermeasures.

During blast exposures, the skull filters out the highest frequency pressure waves, yielding transient intracranial pressures (kPa) dominated by frequencies in the kHz range.^4,5^ Relative to in vivo animal models, the human head/skull may have more susceptibility to produce high intracranial pressures from blast exposures—with resultant intracranial pressure peaks multiple times larger than the incident overpressure.^6^ In addition to transient high intracranial pressures, during incidence of large blasts, there can be concomitant high strain rates from brain acceleration.^7,8^ Given neuronal^9–12^ and^13^ glial sensitivity to strain rates, specific pathologic/physiologic changes to either pressure or deviatoric strain have yet to be defined. However, individuals exposed to LLBs occupationally^14,15^ exhibited concussion-like symptomatology. Deviatoric strain amplitudes associated with LLBs are predicted to be lower than established thresholds for neuronal injury.^5^ Exposed individuals showed reduced reaction time relative to baseline and changes in serum biomarkers, which suggests that transient intracranial pressure could change neural function and cognitive performance. Clinically, electrophysiological disruptions are recorded using electroencephalogram or electrocorticogram, which monitors neural activity or oscillations across brain regions. As these network oscillations are attributed to different cognitive functions including memory,^16^ attention,^17^ executive motor function,^18^ and sleep,^19^ modulations of this activity may function as an electrographic biomarker for TBI and provide insight into its sequelae. Although the concepts of a unique pressure-induced brain injury have been suggested since early biomechanical engineering analysis of head trauma,^20^ the existing, proposed pressure injury thresholds (typically 250 kPa)^5,21^ were derived from cadaveric and animal models under blunt loading^22^ and have not been validated in blast. There is limited experimental data describing the physiologic response to predominately high frequency pressure loading; however, there is limited yet present data suggesting frequency of stress waves may also alter physiology.^23,24^

To isolate physiologic changes from pure quasi-hydrostatic pressure, a unique approach should be taken to address two key challenges: 1) the generation of controlled pressure exposures, and 2) a relevant human-based *in vitro* brain tissue model. Current *in vivo*^25,26^ and *in vitro*^27,28^ blast models utilize spatially and spectrally heterogeneous pressure exposures systems that are not tunable across a range of pressure and frequency parameters. In this study, we utilize a previously developed computer-controlled pressure chamber^29^ to expose human cerebral organoids to varied frequencies of idealized pressure waves that are representative of those experienced intracranially during blast exposure. These cerebral organoids develop the complex cortical architecture with necessary glial and neuronal cell types^30,31^ capable of generating neural network dynamics reminiscent of cortical oscillations.^32,33^ Here, we demonstrate a relationship between pressure wave exposure and neural network dynamics that provide specific parameters to define primary injury during TBI.

## Methods

### Pluripotent stem cell cultures

Human pluripotent stem cells (H9, WA09, WiCell) were cultured feeder-free and maintained on Matrigel™ (BDBiosciences, 354230, growth factor reduced) coated plates in mTeSR-1 medium (Stem Cell Technologies, 05850) plus 1% penicillin and streptomycin (Life Technologies, 15070).

### Generation of cerebral organoids

Cerebral organoids were generated following previously published methods.^30,31^ Briefly, pallial and subpallial spheroids were generated from pluripotent stem (PS) cells and assembled *in vitro* to replicate the development of the human cerebral cortex. Pallial spheroids were generated by plating dissociated PS cells into ultra-low attachment 96 well plates to form embryoid bodies in neuro induction medium (NIM) containing 20 μM ROCK inhibitor (Y-27632, Selleckchem #S1049). NIM contains: DMEM-F12 (Invitrogen) with KnockOut Serum Replacement (20%, Invitrogen), GlutaMAX (1:100, Invitrogen), MEM-NEAA (1:100, Gibco), 2-mercaptoethanol (0.1 mM, Gibco), penicillin and streptomycin (1%, Sigma). Dorsomorphin (10 μM, Sigma) and SB-431542 (10 μM, Tocris) were added for the first 6 days for neural induction. Spheroids were transferred to neural medium (NM) containing Neurobasal A (Gibco, 10888) with B27 supplement (- Vitamin A, Invitrogen), GlutaMAX (1:100, Invitrogen), penicillin and streptomycin (1%, Sigma). For neural progenitor expansion, NM was supplemented daily with fibroblast growth factor (20 ng/mL, Peprotech) and epidermal growth factor (20 ng/mL, Peprotech) for 10 days, then every other day for 9 days. For neural differentiation of pallial spheroids, NM was supplemented with brain-derived neurotrophic factor (20 ng/mL, Peprotech) and NT3 (20 ng/mL, Peprotech) every other day until Day 43. After Day 43, all spheroids were maintained in NM without factors.

For development of subpallial spheroids, were prepared as above. However, beginning at Day 4, Wnt pathway was inhibited by adding IWP-2 (5 μM, Selleckchem) to the NIM and NM media. On Day 12, sonic hedgehog pathway was activated by adding SAG (100 nM, Selleckchem) together with IWP-2 until Day 24. After Day 24, subpallial spheroids were maintained in the same conditions as pallial spheroids.

Cerebral organoids were formed by a fusion of the pallial and subpallial spheroids to mimic development of the human cerebral cortex. On Day 43, pallial and subpallial spheroids were transferred into a single well of a 24 well plate and pushed together. The plate was tilted and placed in the incubator for 5-7 days. Media was carefully changed every 4 days. Once complete cerebral organoid fusion occurred, the 24 well plate was returned to a rotating shaker in the incubator and NM without factors was changed 3 days a week.

### Tabletop “blast” device

Quasi-hydrostatic pressure waves were delivered by a previously developed tabletop “blast” device.^29^ Pressure loading in this device can be considered as quasi-hydrostatic for the following reason: pressure waves travel at the speed of sound in water (∼1480 m/sec), and the length of the pressure chamber is nearly 30 mm. In consequence, any change in pressure takes around 20 μs to completely homogenize inside the chamber. The characteristic frequency of an event of this speed is around 50 kHz. This frequency is 10 times as fast as the fastest frequency used in this study (5 kHz). To introduce high frequency pressure wave onto cerebral organoids, prewarmed and CO_2_ buffered NM was added to the “blast” chamber (3-5 mL). Organoids from a single experimental group were added together into the “blast” chamber where it was then closed and sealed without introducing any air bubbles. The chamber was then installed into the support frame.

A user-defined excitation voltage profile was used to drive a piezoelectric actuator and create a pressure waveform within the chamber at 500 Hz, 3000 Hz, or 5000 Hz frequency for a duration of 8 msec. For the readability, these three frequency levels will be referred hereafter simply as Low group, Mid group, and High group, with the implicit understanding that the terms refer to the abovementioned three loading frequency levels. In this study, this frequency space was examined at “threshold” of 250 kPa (as previously proposed)^5,21^ or “suprathreshold” of 350 kPa pressure wave amplitude. Because the chamber is filled with a nearly incompressible liquid (culture medium), organoids inside are equally exposed to similar over pressure profiles. For “no blast” controls, organoids were loaded and sealed into “blast” chamber, but the actuator was not activated. After exposure (∼30 sec process to load and unload), organoids were removed and plated onto a multi-electrode array (MEA) for functional recordings. The integrated blast overpressure for each pressure wave form was calculated numerically using the trapezoidal rule (Python function *numpy.trapz*).

### MEA recordings and custom analysis

Fused cerebral organoids were plated onto a 24-well MEA plate (24W700/100F-288, Multichannel Systems) previously coated with 10 μg/mL poly-L-ornithin (Sigma, P3655) and 5 μg/mL laminin (Invitrogen, 23017-015). At the bottom of each well of the MEA plate is 12 gold electrodes 100 μm in diameter spaced 700 μm apart. Prior to plating organoids, fresh NM was added to each well and allowed to buffer in 5% CO_2_ for 30 min. MEA recordings were acquired with the Multiwell-Screen Acquisition software (Multichannel Systems, v1.11.7.0). For raw electrographic data, organoids were plated immediately after “blast” and the plate was placed on the MEA for 1 h before the first recording. 37°C incubation temperature and 5% CO_2_ was maintained the entire time. All recordings acquired were sampled at 10 kHz and filtered with a band-pass filter of 1 Hz-3500 Hz for 2 min.

### Event detection

Automatic event detection was performed using the Multiwell-Analyzer (Multichannel Systems, v.1.8.6.0). For single unit events, a bandpass filter from 100 Hz-3500 Hz with a second order Butterworth filter. Single unit events were detected based on 5 standard deviations of estimated noise from each active electrode. For population events, a bandpass filter from 1 Hz-3500 Hz with a second order Butterworth filter was applied and event detection criteria was based on 15 standard deviations of baseline noise. Single unit and population events are reduced in response to bath application of the voltage-gated sodium channel antagonist lithium chloride (data unpublished). For all organoids, active electrodes were defined by visual mapping of contacted channels. Electrodes without contact with organoid were not included in the data set. Event amplitude and frequency were calculated by Multiwell-Analyzer. For visualization, event data was exported to .csv files and plotted using custom Python code with *matplotlib, scipy,* and *numpy* libraries.

### Power spectral analysis

To compute the power spectral density, 5-sec epochs from active channels were exported to .csv files and transformed using a discrete Fourier Transform (*numpy.fft*) and averaged peak power (*scipy.signal.find_peaks*). Signal frequency was visualized in the time domain using the spectrogram function (*scipy.signal.spectrogram*). Computation and analysis were adapted from published neural data repository.^34^ Power data was normalized to the corresponding control group.

### Network synchrony analysis

For network synchrony, 2-sec epochs from two selected channels of unfiltered timeseries data was exported and analyzed using Python code adapted from Mike X Cohen.^35^ A Fourier Transform was performed on the raw data and convolved using a complex Morlet Wavelet convolution (10 Hz). The phase angle was computed for both channels from the phase angle complex convolution (*numpy.angle*). Phase synchronization was calculated by the difference between phase angles of the selected channels. This value is bound between 0 and 1, with values close to 1 indicating greater synchrony.

### Immunohistochemistry

All organoids were fixed in 4% paraformaldehyde overnight and transferred to 30% sucrose in phosphate-buffered saline until sectioned. To section, organoids were embedded in tissue freezing medium and frozen. Serial sections were taken at 16 μm and mounted on a glass slide. All sectioned organoids were stored at -20°C until stained. For immunohistochemistry, all slides were washed with 1 × Tris-buffered saline (TBS) and incubated for 1 h at room temperature in a 3% normal goat serum blocking solution. The primary antibody was incubated on the slide overnight at 4°C. Primary antibodies used in this study include mouse anti-TUJ1 (Santa Cruz, sc-80005; 1:500) and rabbit anti-activated caspase 3 (Millipore Sigma, AB3623; 1:500). The primary antibody was washed with 1 × TBS and the fluorescent secondary antibody was incubated for 1 h at room temperature. The secondary antibodies used in this study were anti-mouse FITC (ThermoFisher, F-2761; 1:500) and anti-rabbit TxRed (Vector, TI-1000-1.5). After the secondary was washed, DAPI (4′,6-diamindino-2-phenylindole; ThermoFisher, D1306; 1:10,000) was added for 5 min to visualize nuclei. Slides were cover slipped with mounting medium and dried before imaging.

### Microscopy and imaging

Fluorescent images were acquired on a widefield epifluorescence (Leica, DM2000) or confocal microscope (Nikon A1). Exposure times were consistent for each channel throughout all samples. For AC3 analysis, area of staining was measured using ImageJ software (v1.53k; National Institutes of Health). Briefly, the pixel area of each organoid was measured. Using built in threshold tools, the pixel area of AC3 staining was measured and subtracted from the total area of the organoid. This percentage is represented in the data as % AC of area.

### Statistical analysis

Data are presented as mean ± standard error of the mean (SEM), unless indicated otherwise. Statistical differences comparing means were analyzed using a two-tailed Student's t-test or analysis of variance (ANOVA) for data with equal variances. Tukey's multiple comparisons test for ANOVA were used to determine difference between groups and is indicated by an asterisk and black bar. Pearson's chi-squared test (Χ^2^) was used for data sets where control data was measured at zero. For all data sets, statistical outliers were excluded using Grubbs' test for outliers. All data was collected and analyzed at one time. All statistics were performed using GraphPad with Prism 8.4.3, following Statistics Guide.

#### Data and code availability

All data supporting the findings in this study are provided within the paper. All additional information will be made available upon reasonable request to the authors. Custom MEA Analysis code was written in Python (v.3.10.1).^36^

## Results

### Modeling “blast” TBI in cerebral organoids

**FIG. 1.**
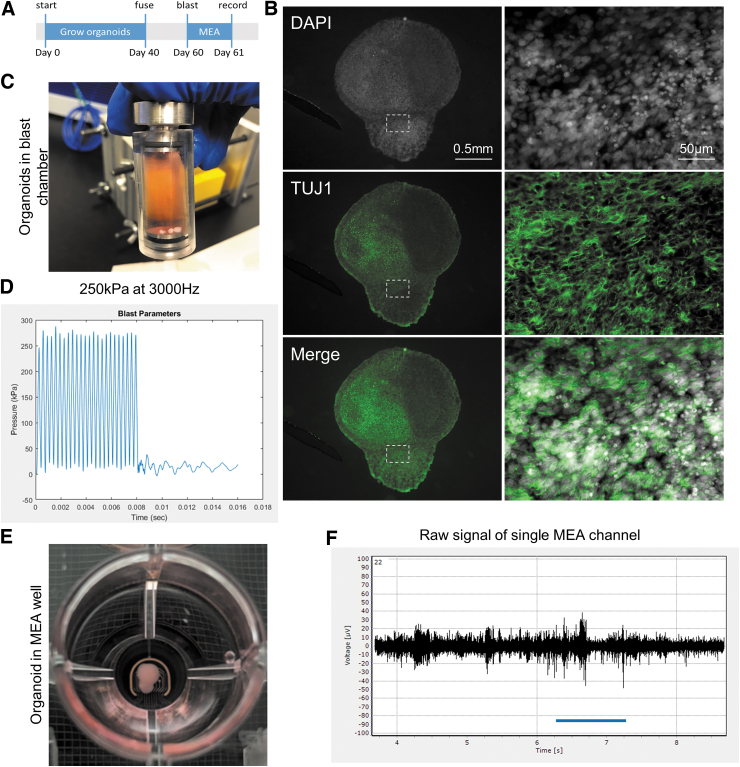
Using tabletop blast device to deliver controlled high-frequency pressure wave injury. **(A)** Pallial and subpallial spheroids were generated from pluripotent stem cells and grown for 40 days before fusing to form a cerebral organoid. Fused organoids were maintained for another 20 days to allow subpallial neurons to migrate and integrate into complex neural networks. At 60 days, organoids were loaded into blast chamber and exposed to pressure waves. Multi-electrode array (MEA) recordings performed made 1 h and 24 h after. **(B)** Representative image of a fused organoid labeled with TUJ1 to visualizes complex neural networks. Dashed box indicates magnified region on the right side of the panel. **(C)** Image of organoids loaded in blast chamber. **(D)** Representative pressure waveform from 3000 Hz frequency that reaches 250 kPa amplitude. **(E)** Image of organoid plated onto MEA. **(F)** Representative trace of raw signal derived from a single channel of the MEA. Color image is available online.

To test the effects of quasi-hydrostatic high frequency pressure waves on cortical circuit function, human pluripotent stem cells were used to grow cortical spheroids that were fused for form fully assembled cerebral organoids (Fig. 1A). These cerebral organoids form complex neuronal networks (Fig. 1B). At 60 days, cerebral organoids were loaded into the tabletop blast device (Fig. 1C) and exposed to high frequency pressure waves with an amplitude around 250 kPa (Fig. 1D). In addition to the “no blast” loaded controls, groups of 12 organoids were exposed to pressure cycles with frequencies from low (500 Hz), mid (3000 Hz), and high (5000 Hz) at a peak pressure of 250 kPa (Supplementary Fig. S1). These parameters were chosen to sample across a broad frequency space of transient intracranial pressures found in blast exposures.^4,5^ Immediately following the exposure, organoids were plated onto an MEA, where recordings were taken at 1 and 24 h post-“blast” (Figure 1E, 1F). At each time point, 12-channels of neural activity were recorded from each organoid.

### Effects of high frequency pressure waves on neuronal activity

**FIG. 2.**
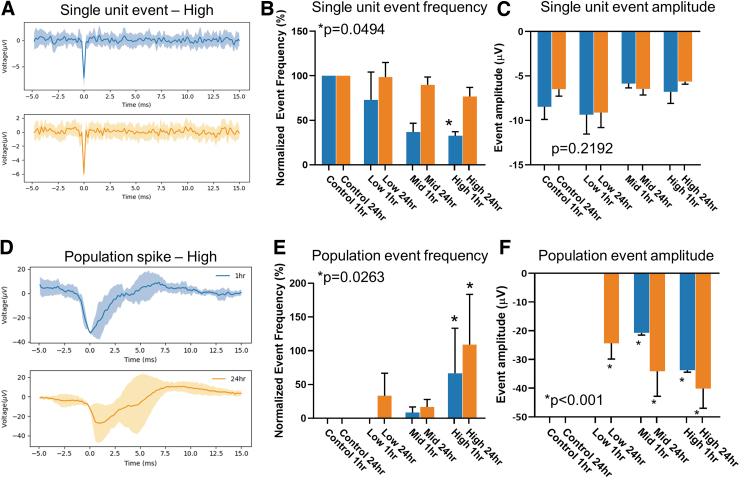
High-frequency pressure blasts acutely changes neural activity of human cerebral organoids. **(A)** Representative traces of single-unit events. In blue (top) the average trace of all events from an organoid exposed to 5000 Hz (High), 250 kPa amplitude pressure wave. In orange (bottom) the average trace from the same organoid at 24 h after exposure. **(B)** Quantification of single unit events normalized to no blast control groups of 1 h (blue) and 24 h (orange) after exposure. **(C)** Quantification of single-unit even amplitudes. **(D)** Representative average trace of population spike event from an organoid at 1 h (blue, top) and 24 h (orange, bottom) after exposure. **(E)** Quantification of population spike event frequency, normalized to control groups. Chi-squared = 15.87, df = 7. **(F)** Quantification of population event amplitude. Error bars presented as standard error of the mean. *p < 0.05, *n* = 12 organoids per group. All statistics calculated using one-way analysis of variance except panel (E), which uses chi-squared test. Color image is available online.

Extracellular action potentials (EAP) recorded using the MEA were characterized by morphology into two groups. The first group appeared to have consistent and reproducible peak amplitude and time-constant (Fig. 2A) between 1 h (top, in blue) and 24 h after exposure (bottom, orange). These EAPs were classified as a single-unit event and were present in all organoids. Compared with “no blast” control organoids, at 1 h following “blast,” the number of these single-unit events decreased as the frequency of pressure wave increased (Fig. 2B). At the lowest frequency tested, there was a modest decrease in single-unit activity to 72.96 ± 31.26% of “no blast” control frequency. At the mid-range frequency, a decrease to 36.67 ± 10% of control, and at highest frequency, there was a reduction in single-unit event frequency to 32.59 ± 4.56% of control. This suppressed single-unit activity returned to 98.54 ± 16.43%, 89.78 ± 8.83%, and 76.64 ± 10.35% of baseline levels within 24 h for low, mid, and high groups, respectively (Supplementary Fig. S2). There was no significant change in the amplitude of these events (Fig. 2C). High frequency pressure waves appear to selectively suppress the firing rate of single-unit events.

The second EAP morphology group was classified as a spontaneous population spike (Fig. 2D; Supplementary Fig. S3), which was characterized by a much larger peak amplitude that varied between events. Unlike the single-unit event, these larger spikes were not present in the controls and only became detectable 24 h after “blast” in the Low group with an increase of 33 ± 33% as compared with the control (Fig. 2E). In the Mid group, an increase of 8.33 ± 8.33% difference was measured 1 h following “blast” that only modestly increased to 16.67 ± 11.24% by 24 h. The High group showed the most dynamic and significant change in population spikes increasing to 66.67 ± 67.8% difference in event frequency (Fig. 2E). Further, as the frequency of pressure wave increased, the peak amplitude of the population spike event also increased (Fig. 2F). In the Low group, population events 24 h after “blast” had an amplitude of -24.35 ± 5.5μV, nearly twice the amplitude of the single-event amplitude (Fig. 2C). In Mid group, the amplitude increased from -20.75 ± 0.75 μV to -34.11 ± 8.71 μV by 24 h. In High group, peak amplitude increased from -33.74 ± 0.69 μV 1 h after “blast” to -40.08 ± 6.88 μV 24 h later. Neither the frequency nor amplitude of the population spikes showed a recovery within 24 h after “blast.” This suggests that pressure waves may target two distinct neuronal populations; one regulating single unit events and one regulating population spikes.

### Effects of high frequency pressure on network oscillations

**FIG. 3.**
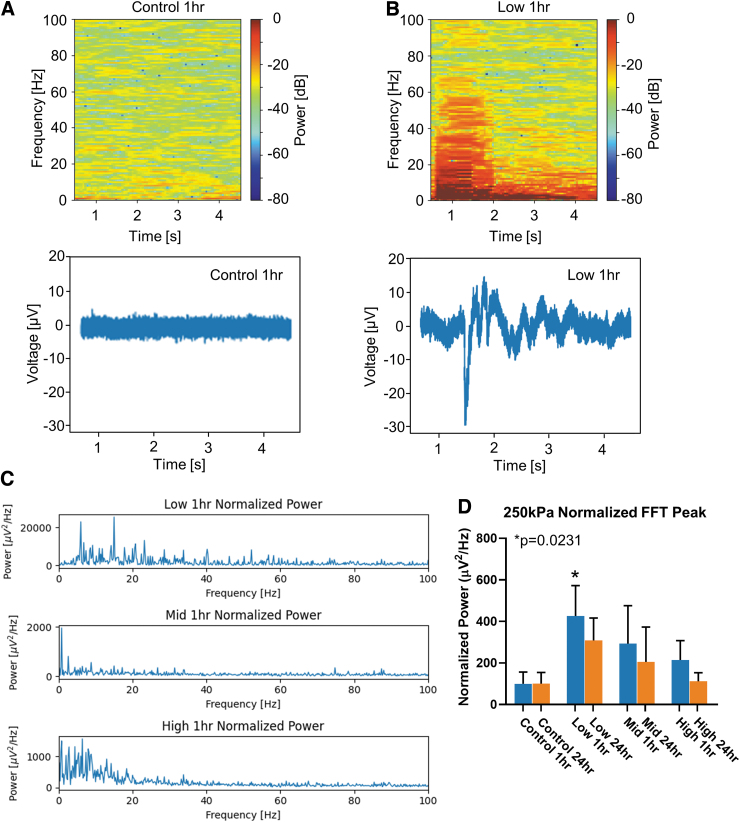
High-frequency pressure blasts enhances network oscillations in human cerebral organoids. **(A)** Top, representative spectrograph from a single channel of a 5-sec epoch at 1 h from a control organoid. Bottom, raw signal from the single multi-electrode array channel. **(B)** Top, representative spectrograph of a single channel at 1hr after exposure to low-frequency pressure wave. Bottom, raw signal that corresponds to the above spectrograph. **(C)** Normalized fast Fourier transform (FFT) of each group. **(D)** Quantification of normalized peak power across all pressure wave parameters tested. Error bars presented as standard error of the mean. *p < 0.05, *n* = 12 organoids per group. All statistics calculated using one-way analysis of variance. Color image is available online.

The acute changes measured in single-unit and population spikes suggest that high frequency pressure waves can interfere with brain function at a cellular level. To determine if neural networks were also altered by high frequency pressure waves, MEA data were analyzed to measure properties of network oscillations (Fig. 2A). A random 5-sec epoch was selected from the 120-sec recording and analyzed across the frequency-time domain. The peak power across time was restricted to the frequency range (0 Hz, 10 Hz). Upon initial visualization of the spectrogram, peak power in this range appeared to change 24 h after “blast” (Fig. 3A, 3B). For each organoid (*n* = 12 per group), the fast Fourier transform (FFT) was computed from each active channel and normalized to the average FFT of the respective control group (Fig. 3C, 3D). The FFT from control organoids in the frequency range (0 Hz, 10 Hz) measured 3.75 ± 2.12 μV2/Hz raw power at 1 h and 0.92 ± 0.50 μV2/Hz by 24 h. The normalized FFT to control power in the Low group measured a significant increase of 425.9 ± 146.5 μV2/Hz at 1 h to 308.0 ± 108.1 μV2/Hz by 24 h. The Mid group had a lower normalized peak FFT of 292.7 ± 183.0 μV2/Hz at 1 h to 204.9 ± 167.8 μV2/Hz by 24 h that was not significantly different than control groups. The High group peak normalized FFT measured 214.3 ± 93.63 μV2/Hz at 1 h and 111.5 ± 41.68 μV2/Hz by 24 h. Only oscillations in the Low group measured a significant increase in power. As the exposure frequency increased, there appears a frequency-dependent threshold for enhancing network oscillations. This suggests low frequency pressure waves alter network function differently than at higher exposure frequencies.

**FIG. 4.**
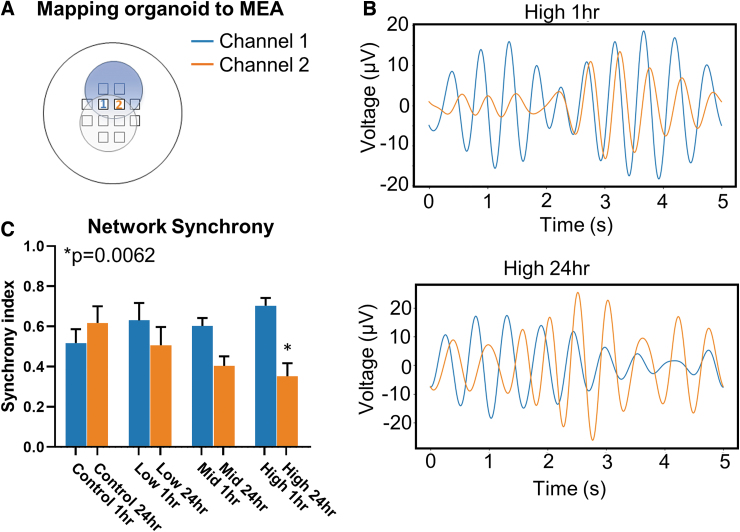
High-frequency blast desynchronizes network oscillations in cerebral organoids. **(A)** Organoids were mapped to multi-electrode array grid to spatially identify channels of interest. For synchrony data, proximal electrodes were selected for active channels to compare synchrony in oscillatory activity. **(B)** Top, filtered oscillations of paired channels from High 1-h exposure group. Channel 1 is presented in blue, and channel 2 is presented in orange. Bottom, similar oscillations from the same organoid 24 h after exposure. **(C)** Quantification of network synchrony across all tested pressure wave frequencies. Error bars presented as standard error of the mean. *p < 0.05, *n* = 12 organoids per group. All statistics calculated using one way analysis of variance. Color image is available online.

Another metric of neural network dynamics is the coherence or synchrony of activity between adjacent populations of neurons. With a 12-channel MEA, spatial and temporal activity can be mapped to measure the synchrony of activity between neighboring channels. Continued activity, propagated in time, between spatial locations suggests functional communication and network coherence. To measure network synchronization, each organoid was mapped to the MEA electrode grid so that specific channels could be identified based on the position and orientation of the organoid on the grid (Fig. 4A). Two neighboring channels (e.g., 1 and 2 depicted) were selected at the fusion plane of the cerebral organoid. A random 5-sec epoch of raw electrographic signal (Fig. 1D) was selected then band-pass filtered with a low-frequency cut-off of 0 Hz and high-frequency cut-off of 10 Hz (Fig. 4B) to calculate the phase angle differences between the two continuous channels. A synchrony index, from 0 to 1, was calculated from the phase angle difference with 0 indicating out of synchrony and 1 indicating perfectly in synchrony. “No blast” controls had a synchrony index of 0.51 ± 0.24 and 0.61 ± 0.08 at 1 h and 24 h, respectively. There were modest, non-significant decreases in synchrony for Low and Mid groups between 1 and 24 h with indices of 0.63 ± 0.09/0.57 ± 0.09 and 0.40 ± 0.05/0.70 ± 0.13, respectively. However, in the High group there was a significant decrease in synchrony index, from 0.70 ± 0.039 to 0.35 ± 0.06 for 1- and 24-h time-points, respectively. This indicates that portions of the filtered signal overlapped in synchrony at 1 h, but by 24 h, there was almost no synchronization within the selected 5-sec epoch (Fig. 4B). Overall, this suggests that at the highest frequency exposure, network oscillations and coherence are disrupted 24 h after “blast.”

### Modeling more larger overpressures on cerebral organoids

**FIG. 5.**
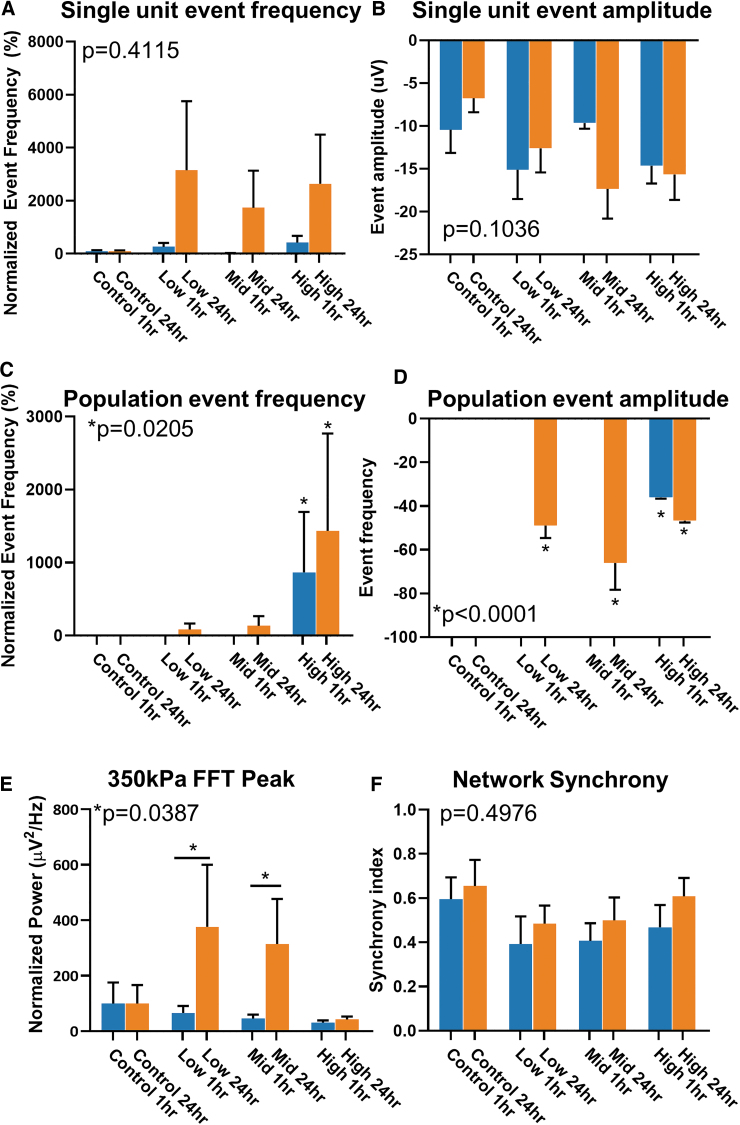
Higher amplitude blast enhances neural activity with no acute recovery. **(A)** Quantification of single-unit event across all pressure wave frequencies tested at 350 kPa amplitude. Data normalized to no blast controls. **(B)**, Quantification of ingle unit event amplitude across all tested frequencies. **(C)** Quantification of population spike event frequency normalized to no blast controls. Chi-squared = 16.55, df = 7. **(D)** Quantification of population spike event amplitude. **(E)** Quantification of peak power from normalized fast Fourier transform (FFT) across all groups. **(F)** Quantification of network synchrony between two channels. Error bars presented as standard error of the mean. *p < 0.05, *n* = 6 organoids per group. All statistics calculated using one-way analysis of variance except for panel (C), which uses chi-squared test. Color image is available online.

To determine if a larger amplitude pressure wave had similar acute effects on the neurophysiology of complex neural networks, another set of cerebral organoids were exposed to the same frequency space (Low, Mid, and High) at a maximal amplitude of 350 kPa. Unlike the lower amplitude blast, there was a drastic increase in single-unit events between 1 and 24 h. Compared with controls, organoids in the Low group had an increase of 265.7 ± 147.5% to 3152 ± 2606% in single-unit event frequencies from 1 h to 24 h after exposure. Similarly, for Mid and High groups, there were increases from 25.32 ± 7.14% to 1738 ± 1396%, and 426.2 ± 249.7% to 2645 ± 1855% over time, respectively (Fig. 5A). In addition to the increase in the exposure frequency of these events, there appears to be no recovery observed at 24 h compared with the changes in lower amplitude “blasts.”

Overall, the amplitude of the single-unit events did not have any significant change between all groups (Fig. 5B). Although population spikes were sparse, like lower amplitude exposures, they seem to be only present 24 h after “blast.” In the Low group, spontaneous population spikes only appeared after 24 h in one organoid at 83.33 ± 84.43% difference from controls. Similarly, in the Mid group, population spikes only appeared at 24 h in a single organoid at 326.6 ± 133.3% difference above controls. In the High group, there was a drastic increase from 866.7 ± 827.3% to 1433 ± 1336% difference (Fig. 5C). The amplitudes of the population spikes were greater than those observed in the lower amplitude group, and were significantly higher between 1 and 24 h. In Low and Mid groups, spike amplitudes increased to -48.97 ± 5.69 μV and -66.0 ± 12.26 μV, respectively, from a 1 h when no events were recorded. In the High group, the populations spike amplitude increased from -36.06 ± 0.53 μV to -46.71 ± 0.83 μV at 1 and 24 h. Further, the drastic increases did not appear to recover after 24 h post-“blast.” The results suggest that “blasts” at higher amplitudes affect neural activity and function differently than those at a lower amplitude.

Network dynamics after higher amplitude “blasts” had different spectral dynamics compared with lower amplitude “blasts.” The normalized peak power, within the (0 Hz, 10 Hz) frequency range, had a similar increase in power to the lower amplitude “blast” (Fig. 3D), however the response was delayed 24 h. In control organoids, the raw peak power at 1 h was 0.1339 ± 0.1 μV2/Hz and 0.122 ± 0.08 μV2/Hz by 24 h. In the Low group, the normalized peak power to control was 65.47 ± 25.67 μV2/Hz at 1 h and significantly increased to 376.5 ± 224.0 μV2/Hz by 24 h. In the Mid group, the normalized peak power was measured at 45.81 ± 13.78 μV2/Hz at 1 h and 314.1 ± 163.3 μV2/Hz by 24 h. In the High group, the normalized peak power was 31.13 ± 7.88 μV2/Hz at 1 h, and 24 h, 42.48 ± 10.31 μV2/Hz. This delayed enhancement in normalized peak power within network oscillations below 10 Hz bandwidth did not change network synchrony across any groups (Fig. 5F). In “no blast” controls, the synchrony index measured 0.59 ± 0.09 at 1 h and 0.65 ± 0.11 at 24 h. In the Low group, the synchrony index was 0.39 ± 0.12 at 1 h and 0.48 ± 0.08 at 24 h. In the Mid group, it was 0.41 ± 0.08 by 1 h and 0.50 ± 0.1 by 24 h. In the High group, the synchrony index was 0.4383 ± 0.10 by 1 h and 0.61 ± 0.08 by 24 h. Unlike the lower amplitude exposure parameters, there was no change in synchrony with higher frequency pressure waves. Altogether, these data suggest that higher amplitude pressure waves change the neurophysiology of complex neural networks differently than lower amplitude pressure, and those changes do not recover over time.

### Injury pattern in cerebral organoids following high frequency pressure

**FIG. 6.**
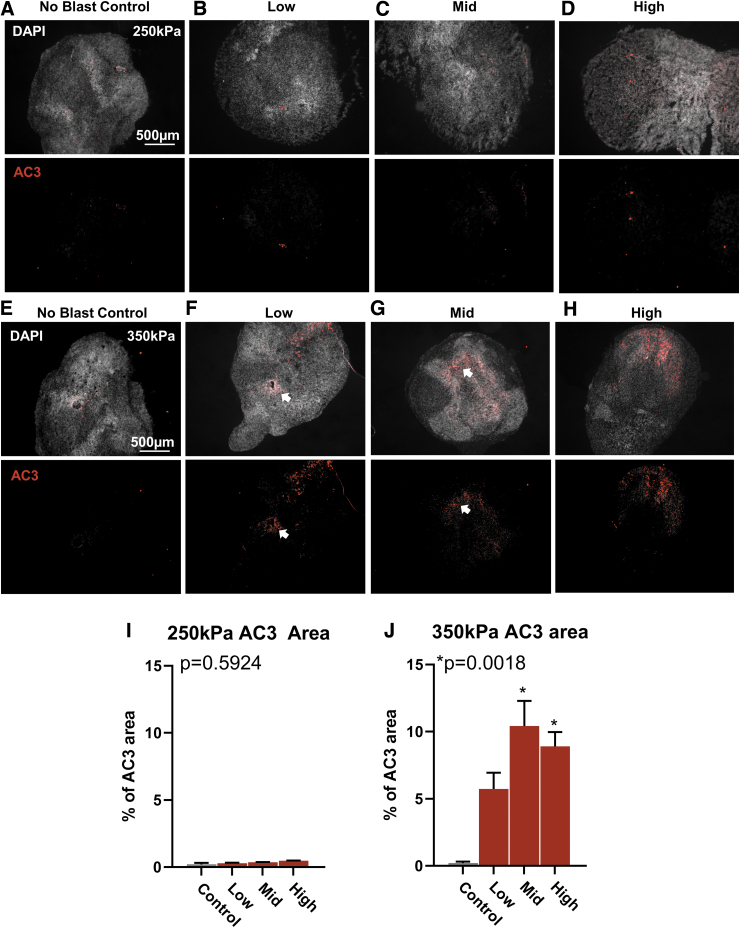
Higher amplitude blast increases cellular damage. **(A)** Representative image of no blast control organoid. Top, individual cell nuclei stained with 4′,6-diamindino-2-phenylindole. Bottom, immunohistochemistry for activated caspase 3 (AC3) to indicate cellular damage and programmed cell death. **(B–D)** Representative histology of an organoid exposed to Low, Mid, and High at 250 kPa amplitude pressure, respectively. **(E–H)** Representative histology of organoids exposed to Low, Mid, and High, at 350k Pa. **(I)** Quantification of AC3 area from organoids exposed to all frequencies at 250 kPa. **(J)** Quantification of AC3 area from organoids exposed to all frequencies at 350 kPa. All data presented as percent (%) area of AC3 to organoid area. Error bars presented as standard error of the mean. *p < 0.05, *n* = 6-12 organoids per group. All statistics calculated using one-way analysis of variance. Color image is available online.

To determine if exposed cerebral organoids demonstrate similar patterns of localized injury, histological sections were taken, and the area of activated caspase-3 (AC3) expression was measured for each organoid as an indicator of apoptosis. As expected, for “no blast” controls across all exposure groups, very few cells were found to express AC3 throughout the organoid (Fig. 6A, 6E). For the exposure group at 250 kPa amplitude, the percent of AC3 area was comparable to controls across increasing pressure wave frequency (Fig. 6B–D, 6I). The cells did not appear localized to any correlated region and were characterized as diffusely patterned. Conversely, for high amplitude exposure group at 350 kPa, there was an observed increase in the area of AC3 expression with all exposed groups (Fig. 6F–H, 6I). The pattern of expression was also localized to regions higher in cellular density as observed by brighter DAPI staining, indicating compact cellular regions. AC3 positive cells were also found localized around tubule shaped ventricular regions (Fig. 6F and 6G; arrow). All together, these results suggest that lower amplitude pressure waves at all frequencies do not elicit observable cellular injury, but with increased amplitude, diffuse cellular injury is apparent with possible co-location in regions of highest density.

## Discussion

Complex forces exerted by primary blast pressure waves on brain tissue is likely heterogeneous and modulate neurological functions. In this study, cerebral organoids from pluripotent stem cells were grown as a standardized *in vitro* model of complex neural circuits that replicate the cortical niche. These organoids develop complex neural circuits capable of generating network oscillations reminiscent to cortical brain waves. To delineate how pressure alters complex neural circuits, a tabletop high frequency pressure “blast” device^29^ was used to deliver controlled pressure waves with two different amplitudes across multiple frequencies. Overall, the data suggests that specific parameters of high frequency pressure exposure can alter neurophysiological properties.

In response to pressure waves at 250 kPa, cerebral organoids demonstrated frequency-dependent suppression of single unit event frequency 1 h after exposure to increasing pressure wave frequencies. These single unit events appear consistent in amplitude and morphology suggesting they are extracellular action potentials generated by individual neurons located near the electrode. This suggest that high frequency pressure waves briefly suppress individual neuronal activity. In addition to simple single unit modulations, larger complex shaped events were also identified and only present after blast exposure, which suggest that groups of neurons were recruited and synchronously activated. The amplitude of these population spikes increased as the frequency of pressure wave increased. The dichotomy of single unit frequency suppression with increased population events could be due to the nuanced differences between neuron populations in response to pressure wave transmission through organoid tissue. Further targeted studies will need to be performed to quantify neuron population response differences to high frequency pressure.

### Pressure waves on neural network dynamics

Cortical function is maintained by a balance of glutamatergic and GABAergic neurons that are excitatory and inhibitory in function, respectively. With focal TBI patients, where tissue damage is prevalent, there is a common increase in extracellular glutamate response.^37^ In severe TBI rodent models, extracellular GABA has been similarly measured to decrease acutely following TBI.^38^ The cerebral organoids in this study consists of both excitatory and inhibitory neurons, which is accomplished by the recapitulation brain development by fusing one spheroid patterned for dorsal pallium development (excitatory neurons) to the ventral pallium spheroid (inhibitory neurons). Fusion of two spheroid allows self-integration of mature inhibitory and excitatory neurons into complex cortical circuits.^30^ The distribution of excitatory and inhibitory neuronal populations may account for dichotomized response in neurophysiology after exposure. Where individual inhibitory neurons may be localized on the surface, the single-unit events may be extracellular action potentials of inhibitory neurons. In response to high frequency pressures, a cascade of reduced inhibition may remove suppression of excitatory populations that are only measured once they are recruited together. Lower amplitude, high frequency pressure waves may selectively suppress neuronal populations that have global neural network effects.

With the pressure waves at 250 kPa, the observed acute changes typically recover to baseline within 24 h, as demonstrated with network oscillations below 10 Hz and the decoupling of these oscillations between electrodes. As excitatory neurons release more glutamate, this would recruit and activate more neurons locally. A well-established primary mechanism following TBI is the increasing influx of calcium via extracellular channels and intracellular stores. In excess, this is known to promote excitotoxicity, cell death, and chromatin remodeling.^39^ With 250 kPa pressure waves, it is possible that calcium channels may be selectively altered in neurons to enhance calcium-dependent vesicle exocytosis (i.e., neurotransmission). This further suggest that discrete population of neurons in the brain may respond differently to pressure waves depending on network confirmation and organization.

As neuronal populations increase oscillatory activity in the <10 Hz range, there was decoupled synchrony between neighboring populations at 24 h post-“blast.” This desynchronization was only significantly different at the higher 5000 Hz waveforms, indicating that the tissue frequency response may have a threshold. In the cortex, neural network oscillations in the low frequency range (< 10 Hz) can be generated by subthreshold membrane potential oscillations that are mediated by hyperpolarization-activated cyclic nucleotide-sensitive (HCN) channels.^40,41^ These subthreshold membrane oscillations were shown to be modulated by parvalbumin interneuron inhibition.^42^ Further, theta oscillations in this bandwidth have been implicated in mild TBI studies both in rodent models^43^ and patients.^44^ High frequency pressure waves (5000 Hz) may decouple network synchronization by disrupting synaptic transmissions between inhibitory interneurons and excitatory cortical neurons. Altogether the results indicate that pressure waves may selectively target specific neuronal populations, perhaps GABA interneurons, additional studies would be necessary to delineate specific neuronal population responses from one another.

Organoids exposed to higher amplitude overpressures (350 kPa) overall responded in a drastically different way. No neurophysiological response measured recovered within 24 h. This may result in chronic changes that the lower overpressure did not induce. This was confirmed with activated caspase 3 activity of apoptosis. Interestingly, cell death appeared to increase as a function of pressure wave frequency. The highest frequency and largest amplitude pressure parameters (350 kPa at mid and high frequencies) induced the most significant cell death. The increased amplitude may still selectively target discrete populations but result in cellular death.

Pressure waveform peak amplitudes for this study have previously been characterized as mild (250 kPa) to moderate (350 kPa) in rodent TBI models.^45^ Using activated caspase 3 (AC3), an upregulated protein during programmed cell death, we found no evidence of apoptosis under lower amplitude “blast” parameters, which was consistent at all frequencies. Similar overpressure amplitudes on isolated neuronal cultures have been reported with in changes in axon beading with little changes in viability with a single blast.^28^ Additionally, in *ex vivo* hippocampal slices exposed to mild TBI overpressures have also shown axon beading, which suggests isolated neurons and reduced circuit preparations respond to mild pressure changes.^27^ However, in both studies, tissue shear stress could not be eliminated, was not quantifiable, and the pressure waveform was more complex and multi-modal.^27,28^ An additional advancement with our organoid approach is that the complex cytoarchitecture of a complex neural circuit is maintained without introducing confounding slice injury necessary to assess in rodent brain slice cultures. These lower amplitude exposures do not appear to injure cells and together with the acute changes in neurophysiology suggest that 250 kPa amplitude waveforms across 500 Hz-5000 Hz frequencies induce a disruption in brain function.

### Pressure waves on cell physiology

It remains a very interesting and open question on the mechanism by which pressure waves alter physiology. There exists a robust literature describing that ultrasound induces changes in cell physiology and structure, even in settings where cavitation is not induced.^46^ Multiple hypotheses have been proposed to include oscillatory movement of intracellular structures, formation of gas bubbles within bilayer cell membrane, and disruption of cytoskeletal elements. Although the employed overpressures in our experiments are of similar amplitude to previous ultrasound exposures (in the 100s of kPa), other key differences also exist, perhaps limiting the utility of previous work to understand the physiology of primary blast exposure. Firstly, the frequencies of exposures in this study were 5 kHz or less, while ultrasound studies are usually in the MHz range. Secondly our exposures do not have any negative pressure phase, whereas ultrasound exposures usually have a symmetric negative and positive pressure. Lastly the presented exposures occurred over only 8 msec while most ultrasound exposures are on the order of minutes.^46^

Additional hypotheses for mechanism also arise from a sparse set of literature quantifying the cellular and subcellular response to high static pressures—usually in the context of deep-sea organisms surviving under high hydrostatic pressures. Static pressures on the order of 100s of MPa's will depolymerize microtubules,^47^ may interfere with the membrane structure and denaturation of proteins in baking yeast,^48^ or alter the packing of cellular membranes leading to conformational changes of transmembrane proteins.^49^ A proposed mechanism for microtubule sensitivity to high pressure is secondary to packing voids within microtubules which compress under hydrostatic pressure^50^—findings most clear in the 100 MPa range. However, a more general theory for protein sensitivity to pressure has been presented and was supported by molecular dynamics simulations of aqueous proteins at physiologically relevant pressures of ∼100 kPa. This theory proposes that at higher pressures proteins can denature to conformations with reduced volume of voids.^51^ However, testing the applicability these previously proposed cellular responses to pressure to the cytoarchitecture of cortical neurons would require additional targeted investigations.

### Limitations

There are a few limitations to this effort. First, cerebral organoids have not yet been an established model of traumatic brain injury. The complex cortical niche of cerebral organoids offers a more sophisticated *in vitro* brain model compared with slice preparations and isolated neuronal culture; however, self-organizing cerebral organoids currently lack vasculature and immune lineage cells (e.g., T-cells/B-cells), both paramount to secondary and tertiary injury responses. Given the presence of neuronal and glial lineage cells, cerebral organoids are poised to study direct effects of mechanical strain on complex neural networks. Further studies that link the neurophysiological changes to molecular changes are needed to support and offer insight into underlying mechanisms of TBI.

Second, the pressure exposures have some limitations. Computer simulations^52,53^ and human phantom modeling suggest during blast exposures intracranial pressure cycles are composed by a mixture of multiple frequencies^54^—with a majority of power contained in the range up to 5 kHz—and decay on the order of 10 msec. Thus, the loading cycles chosen in this study do not represent pressure cycles specific to any blast exposure scenario. Due to the complexity of intracranial pressure cycles, that loading space is basically infinitely large. Hence, the loading cycles chosen herein are strategic discrete samples of the frequency spectrum and amplitude of pressure cycles representative of blast exposure. The six different loading scenarios were chosen to test if frequency or amplitude of intracranial pressure waves would incite any patterns of cellular dysfunction or damage. An additional advantage to our chosen waveforms is that the time average of the pressures for each scenario were grossly equivalent between different harmonic loadings.

## Conclusions

Although this study was not designed to isolate specific mechanisms by which pure pressure would alter cellular physiology, it does provide evidence of a dose dependent (in amplitude and frequency) tissue response in cerebral organoids to high pressure waves. The exposures presented here are idealized, but representative of the spectrum and duration of pressure waves experienced intracranially during blast exposures. In this preliminary study, lower pressure (250 kPa) waves induce an acute neurophysiological response which appears to alter physiology without significant cellular destruction. Larger amplitude pressure waves (∼350 kPa) appear to induce cellular death, permanent alterations in physiology with possible frequency dependent response. Further work must be done to reproduce such findings, and if robust targeted investigations are warranted to understand the physiologic and molecular mechanism of these changes. A key strength of this approach will be the ability to precisely test parameter space via a computer-controlled pressure chamber. However idealized, this approach may provide a new tool to define a threshold of TBI injury from primary blast exposure, explore cellular and subcellular responses to blast, and possibly be a test bed to trial therapeutics for primary blast exposure.

## Supplementary Material

Supplemental data

Supplemental data

Supplemental data
