# Supplementary material for: Understanding Primary Blast Injury: High Frequency Pressure Acutely Disrupts Neuronal Network Dynamics in Cerebral Organoids
Source: J Neurotrauma. 2022 Nov 1;39(21-22):1575–90. doi: 10.1089/neu.2022.0044 (PMC9689772; doi:10.1089/neu.2022.0044)
Supplement: Supplemental data [file Supp_FigureS1.docx]

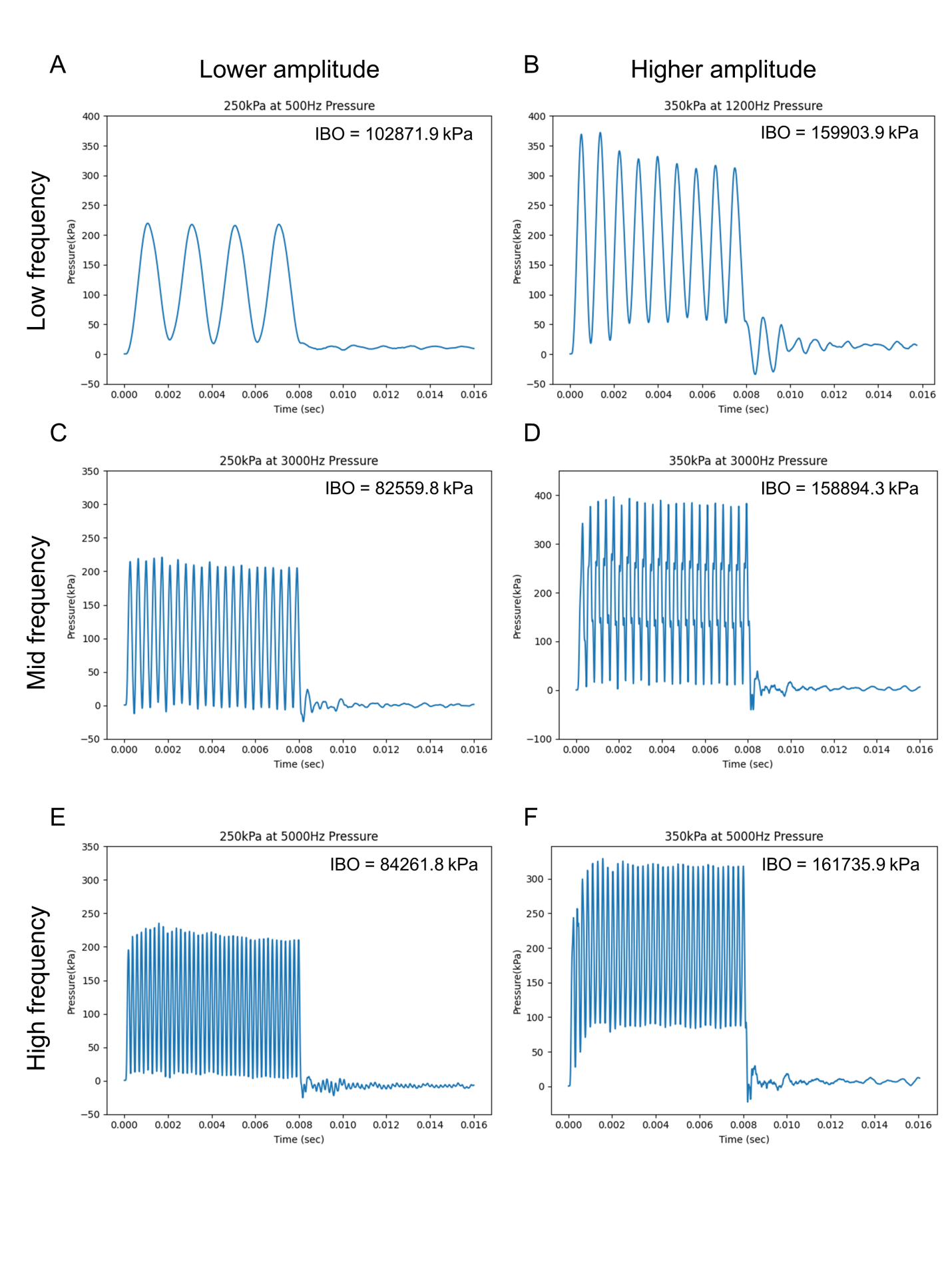
Supplemental Figure 1. Representative pressure waveforms generated from table-top device. A, Representative pressure wave measured within the table top device for a lower amplitude (<250kPa) low frequency (500Hz) waveform. B, Higher amplitude (350kPa), lower frequency (1200Hz) waveform. C, Lower amplitude, mid frequency (3000Hz). D, Higher amplitude, mid frequency. E, Lower amplitude, higher frequency. F, Higher amplitude, higher frequency. For each pressure wave form, the integrated blast overpressure (IBO) is displayed.
