# Supplementary material for: Understanding Primary Blast Injury: High Frequency Pressure Acutely Disrupts Neuronal Network Dynamics in Cerebral Organoids
Source: J Neurotrauma. 2022 Nov 1;39(21-22):1575–90. doi: 10.1089/neu.2022.0044 (PMC9689772; doi:10.1089/neu.2022.0044)
Supplement: Supplemental data [file Supp_FigureS3.docx]

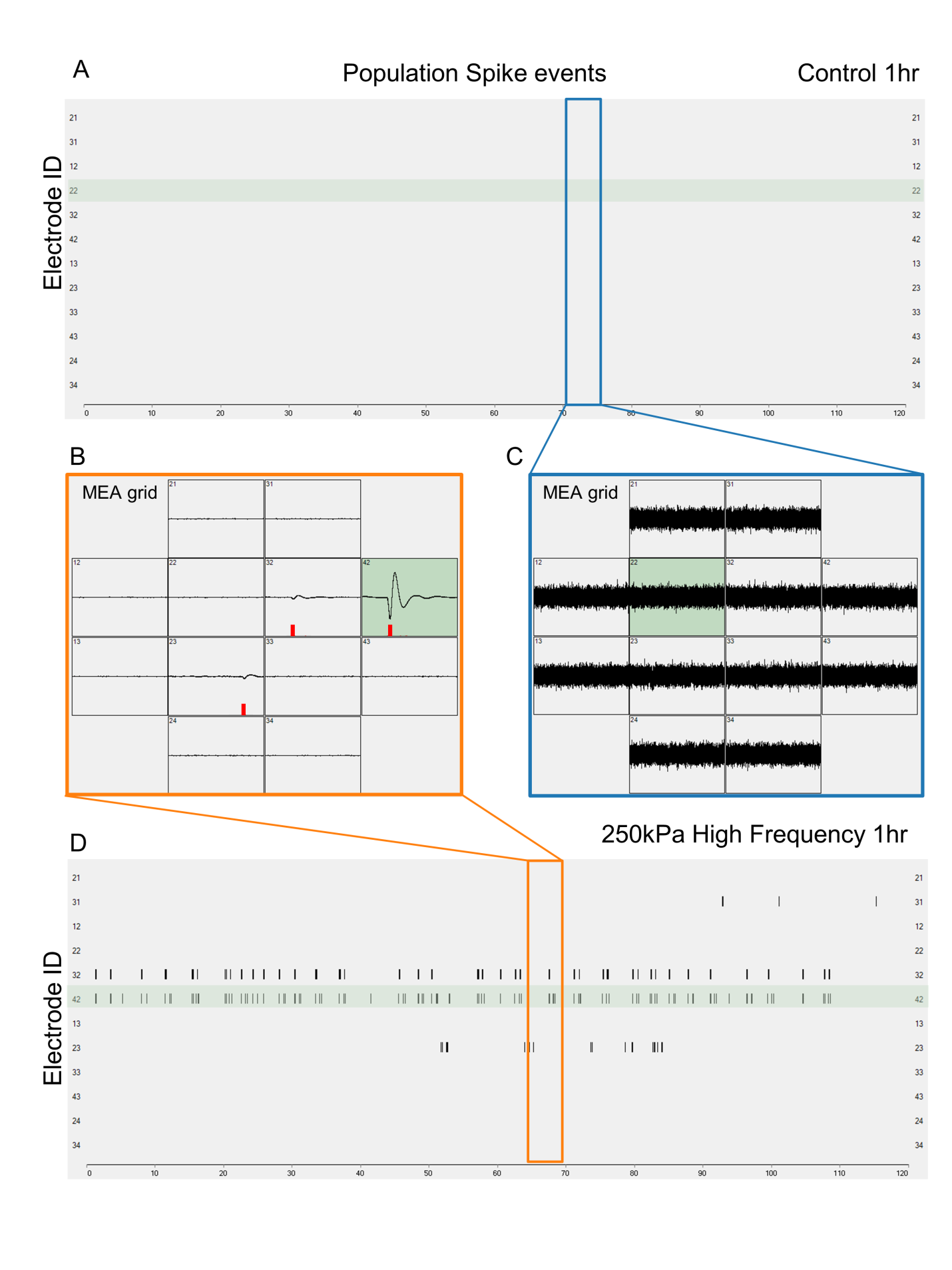
Supplemental Figure 3. Raster plots for population spike events across entire MEA well. A, Representative raster plot from a control organoid. Each row represents a single channel from the MEA with the electrode ID indicated along the side panel. Each dash represents the detection of an event. Green band highlights the corresponding channel found the MEA grid panel. B, Raw traces highlighted by the color window on the raster plots (orange represents the example after exposure, blue is from the control). Each red line is the single unit event indicated by the raster plot. Each MEA grid corresponds to a 5 second epoch with a +/- 400µV scale. Electrode ID is located in top left corner and corresponds to the electrode ID of the raster plot. C, Representative MEA grid from the control organoid, each MEA grid corresponds to a 5 second epoch with a +/- 12.5µV. D, Raster plot from an organoid following exposure to 250kPa, high-frequency pressure wave.
